# A Report of a Rare Case of Kounis Syndrome in a Patient With HIV/AIDS and Mpox

**DOI:** 10.7759/cureus.63957

**Published:** 2024-07-06

**Authors:** Vivek Jha, Saroj K Jha, Badri Aryal, Swati Dhungel, Supriya Jha, Max Brock

**Affiliations:** 1 Internal Medicine, John H. Stroger, Jr. Hospital of Cook County, Chicago, USA; 2 Internal Medicine, Tribhuvan University Institute of Medicine, Kathmandu, NPL; 3 Cardiology, John H. Stroger, Jr. Hospital of Cook County, Chicago, USA

**Keywords:** vancomycin induced kounis syndrome, allergy and anaphylaxis, acute coronary syndrome (acs) and stemi, st-elevation myocardial infarction (stemi), kounis syndrome

## Abstract

Kounis syndrome (KS) is defined as the occurrence of acute coronary syndrome due to coronary artery spasm in a patient with an allergic reaction. Antibiotics are the most common trigger for KS. In this case report, we present a 45-year-old man with HIV/AIDS who was being managed for mpox and developed chest pain and hypotension during vancomycin infusion, which was complicated by the development of ST-elevation myocardial infarction (STEMI). His left heart catheterization showed normal coronaries with the resolution of ECG changes and symptoms upon discontinuing vancomycin.

## Introduction

Kounis syndrome (KS), an illness initially identified by Kounis and Zavras in 1991, is characterized by myocardial ischemia brought on by coronary artery spasm in association with an allergic or anaphylactic response [1]. It is often referred to as "allergic myocardial infarction" or "allergic angina syndrome." During an allergic reaction, mast cells release proinflammatory mediators such as histamine that act on receptors in the heart and coronary arteries to cause this condition [2]. Various environmental exposures and medications, including antibiotics, can trigger KS. Clinically, it presents with symptoms and signs suggestive of acute coronary syndrome (ACS), which rarely correlate with findings on coronary angiography. Due to its presentation alongside an allergic manifestation, it poses a diagnostic dilemma and a therapeutic challenge, necessitating increased awareness of KS among physicians.

We present a case report of a 45-year-old male with HIV/AIDS and mpox (formerly known as monkeypox) who developed KS following vancomycin administration. This case report aims to focus on the characteristics of KS and highlight the peculiar challenge in its diagnosis and management.

## Case presentation

Our case is of a 45-year-old male with HIV/AIDS who was referred to the emergency department (ED) from the HIV clinic for evaluation of mpox and concern for meningitis. He presented with headache, dysuria, rash, fever with chills, night sweats, neck pain, and stiffness for two days. He had a past medical history of aseptic meningitis and neurosyphilis (which were successfully treated) along with poorly controlled HIV/advanced AIDS (CD4 count of 6) for which he had not taken antivirals for the last four months.

On presentation, he was afebrile and had stable vitals with a blood pressure (BP) of 142/82 mmHg, a pulse of 105 beats per minute, a respiratory rate of 23 breaths per minute, and oxygen saturation of 98%. A physical exam revealed multiple rashes, especially at the base of his penis, scattered lesions on his arms, multiple firm vesicles on his face and lips, and a few white flat lesions on the palate, suggestive of mpox. Hematologic and biochemical lab tests were within normal limits. Hepatitis A IgG, Hepatitis C antibody, and COVID-19 PCR were nonreactive. ECG was normal with sinus rhythm. CT head showed no intracranial abnormality, after which a lumbar puncture was done due to concern for aseptic meningitis. Ceftriaxone and vancomycin were started in the ED due to a concern for bacterial cellulitis. The infectious disease service was consulted, and the patient was started on empiric treatment for mpox with acyclovir and tecovirimat.

**Figure 1:**
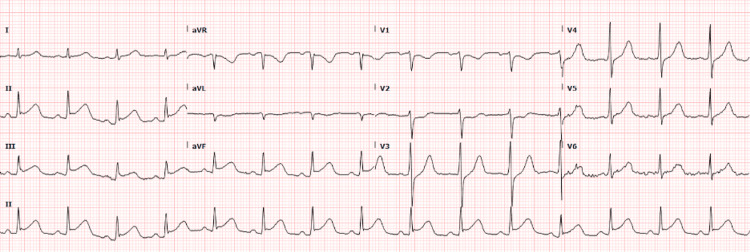
ECG showing ST-segment elevation in leads II, III, and aVF, and reciprocal ST depression in leads V1-V3.

**Figure 2:**
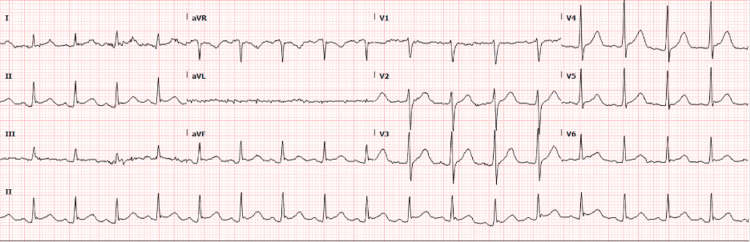
Repeat ECG showing resolution of ST-segment elevation in inferior leads and resolution of ST depression in V1-V3.

During vancomycin infusion, he developed acute central chest pain, hypotension (BP: 71/44 mmHg), and tachycardia. ECG was obtained and showed new ST-segment elevation in leads II, III, and aVF, and reciprocal ST depression in V1-V3 suggestive of inferior wall ST-elevation myocardial infarction (STEMI) (Figure 1). Troponin level was elevated; initial troponin was 0.461 (normal range: 0.000-0.039) at the time of chest pain and repeat troponin after seven hours was trending at 5.241. Repeat ECG after 15 minutes showed continued ST-segment elevation. He briefly required norepinephrine for his hypotension which resolved. He was given aspirin 325 mg, a high-intensity statin and vancomycin was discontinued. Urgent left heart catheterization (LHC) was pursued, which showed normal left dominant circulation, no obstructive lesions, and no evidence of current spasms. Repeat ECG following LHC was normal with the resolution of ECG changes (Figure 2); hence, aspirin was discontinued. At that point, important differentials we considered were myocarditis, Takotsubo cardiomyopathy, and coronary artery spasm secondary to vancomycin reaction (KS).

After the resolution of symptoms, an echocardiogram was done which ruled out Takotsubo cardiomyopathy. Echo showed low-normal systolic function with an ejection fraction of 52% and no regional wall motion abnormalities. Complete blood count demonstrated new onset hypereosinophilia, rising from 0.9% on admission to 8.6% (absolute count of 500) on the day of chest pain. The patient had no history of variant angina and was diagnosed with KS based on the temporal correlation of vancomycin administration, the onset of chest pain, ST-segment elevation in the ECG, increased troponins, hypereosinophilia, and normal coronary arteries on cardiac catheterization with remission of symptoms with anti-ischemic medication and vancomycin discontinuation. Although tryptase was within normal limits, it has to be noted that it was collected three hours after the event when KS was suspected retrospectively. Later, cardiac magnetic resonance imaging (CMR) showed evidence of mild mapping abnormalities (mild elevation in the T1 and ECV in the mid and basal levels of LV) in a vascular distribution consistent with the right coronary artery (RCA).

Concurrently, polymerase chain reaction (PCR) for the mpox (MB1) virus was positive and he continued to improve with treatment for mpox and HIV/AIDS. He was discharged with advice to continue taking HAART for HIV/AIDS, and tecovirimat for mpox with a follow-up appointment scheduled with the HIV clinic.

## Discussion

The co-existence of ACS in patients with allergic reactions is referred to as KS [3]. In our case, the patient's chest discomfort started soon after the vancomycin infusion. Though he became hypotensive, there were none of the typical allergic/anaphylactic symptoms, including urticaria, pruritus, angioedema, congestion, and wheezing, which is in line with previous case reports of vancomycin-induced KS [4]. STEMI was the initial diagnosis based on presentation, ECG, and troponin values, but left heart catheterization was not consistent with an acute, plaque rupture MI and it revealed non-obstructive coronaries. Diagnosing vasospastic angina can be supported by provocative testing during an invasive coronary angiography. However, that was deferred due to the presence of considerably raised troponin levels and ST-segment elevation.

After the normal coronary angiography, the differential included Takotsubo cardiomyopathy and myocarditis [5]. There were no focal wall motion abnormalities on echo suggestive of Takotsubo cardiomyopathy. Myocarditis can have ECG changes of Q waves and regional ST elevations [6]. Myocarditis-related CMR findings include both T1- and T2-based criteria, such as high T2 signal intensity in a given region or globally, an increase in myocardial T2 relaxation time in a given region or globally, an increase in native myocardial T1 relaxation time in a given region or globally, and a region of elevated T1 that extends beyond the region of late gadolinium enhancement (LGE) [7]. CMR in our patient was inconsistent with these findings of myocarditis and had a mild elevation in the T1 and ECV in the mid and basal levels of LV (RCA vascular territory), without any evidence of myocardial edema or LGE.

There are three main types of KS. Type I affects those who do not already have coronary artery disease, as in our patient. Type II affects individuals who already have coronary artery disease. Type III concerns thrombosis of the stent brought on by inflammatory mediators [8].

An increasing array of factors, such as different diets, medications, environmental exposures, insect bites, and intravenous contrast materials, may trigger KS. Antibiotics account for 27.4% of cases, making them the most prevalent triggers, followed by insect stings (23.4%) [3,9]. Among antibiotics, the majority of the cases are of beta-lactam antibiotic-induced KS and a few case reports of vancomycin-induced KS [10,11].

Proinflammatory mediators and chemokines are released upon mast cell degranulation, causing allergic responses and coronary artery spasms in Kounis Syndrome [2]. The release of histamine, tryptase, chymase, leukotrienes, and cytokines causes endothelial dysfunction, platelet activation, coronary vasoconstriction, and potentially myocardial ischemia or infarction [3,5].

Cardiac enzymes like CK, and especially CK-MB, are useful in identifying cardiac injury brought on by allergic or anaphylactic reactions. Tryptase is another marker of anaphylaxis which is primarily released from mast cells [12]. It has a short half-life of about 90 minutes. The optimal time for measuring tryptase levels is around half an hour after the initial symptoms, with subsequent measurements every 30 minutes for the following two hours [3]. The delayed collection of tryptase after three hours may have resulted in a normal tryptase value for our patient.

Electrocardiography (ECG) typically reveals ST-T changes indicative of ischemia, with STEMI being the most common presentation. A review reported the RCA as the culprit in 50% of the cases [9]. Other ECG changes associated with KS include atrial fibrillation, heart block, nodal rhythm, sinus bradycardia, sinus tachycardia, T-wave flattening and/or inversion, QRS complex prolongation, QT segment prolongation, ventricular ectopics, and ventricular fibrillation. Additionally, localized wall motion anomalies in the afflicted artery's distribution might be seen on an echocardiogram. CMR is also effective in evaluating cardiac involvement in KS, with delayed contrast-enhanced images showing normal washout in the subendocardial lesion area characteristic of type I KS [3,13].

No consensus guidelines exist for treating KS, but the immediate cessation of the offending agent is universally emphasized to minimize further cardiac injury [14]. Treatment involves addressing both the allergic reaction and the cardiovascular consequences, potentially using antihistamines, corticosteroids, and vasodilators like nitroglycerin in severe cases. Current guidelines for ACS do not specifically address KS. Therefore, patients presenting with ACS should be treated according to existing ACS guidelines [5,9].

The prognosis varies based on the severity of the allergic reaction and coronary involvement. With timely recognition and treatment, most patients fully recover which highlights the importance of increased awareness among treating physicians [5]. Serious complications are uncommon, with cardiogenic shock occurring in 2.3% of cases, cardiac arrest in 6.3%, and a death rate of 2.9% [9,15].

## Conclusions

Coronary vasospasm from antibiotics, including vancomycin, is an uncommon but serious reaction. KS should be suspected in ACS patients who exhibit allergic reactions following antibiotic use, especially if they respond poorly to conventional anaphylactic treatment. Effective treatment includes addressing the allergic reaction with prompt cessation of the offending agent and possible myocardial revascularization. Early identification and appropriate anti-ischemic, anti-thrombotic, and anti-allergic treatments can improve patient outcomes. The prognosis for KS is generally better due to early intervention and known allergy triggers; however, there is a lack of data on long-term follow-up and outcomes.
